# Magnetic Nanoparticle-Based Biosensing Assay Quantitatively Enhances Acid-Fast Bacilli Count in Paucibacillary Pulmonary Tuberculosis

**DOI:** 10.3390/bios8040128

**Published:** 2018-12-12

**Authors:** Cristina Gordillo-Marroquín, Anaximandro Gómez-Velasco, Héctor J. Sánchez-Pérez, Kasey Pryg, John Shinners, Nathan Murray, Sergio G. Muñoz-Jiménez, Allied Bencomo-Alerm, Adriana Gómez-Bustamante, Letisia Jonapá-Gómez, Natán Enríquez-Ríos, Miguel Martín, Natalia Romero-Sandoval, Evangelyn C. Alocilja

**Affiliations:** 1 Health Department, El Colegio de la Frontera Sur (ECOSUR), San Cristobal de Las Casas, Chiapas 29290, Mexico; naabani@gmail.com (C.G.-M.); agv23@yahoo.com (A.G.-V.); hsanchez@ecosur.mx (H.J.S.-P.); 2 The Network GRAAL (Grups de Recerca d’America i Africa Llatines)-ECOSUR Node, San Cristobal de Las Casas, Chiapas 29290, Mexico; Miquel.Martin@uab.es (M.M.); nromero@internacional.edu.ec (N.R.-S.); 3 Global Alliance for Rapid Diagnostics. Michigan State University, East Lansing, MI 48824, USA; giuseppemj@gmail.com (S.G.M.-J.); alliedbgraal@gmail.com (A.B.-A.); ernatan@gmail.com (N.E.-R.); 4 Nano-Biosensors Laboratory, Department of Biosystems and Agricultural Engineering, Michigan State University, East Lansing, MI 48824, USA; kpryg22@gmail.com (K.P.); shinner1@msu.edu (J.S.); nathan.h.murray@gmail.com (N.M.); 5 Mycobacteriology Laboratory, TB Prevention and Control Program for the Highlands of Chiapas, Chiapas 29250, Mexico; 6 State Public Health Laboratory for Chiapas, Tuxtla Gutierrez, Chiapas 29040, Mexico; adrgomezb73@hotmail.com (A.G.-B.); letisiajgo@hotmail.com (L.J.-G.); 7 Communicable and Non-communicable Diseases Department, Ministry of Health of Chiapas, Tuxtla Gutierrez, Chiapas 29010, Mexico; 8 Biostatistics and Epidemiology Unit, Autonomous University of Barcelona, 08193 Bellaterra, Spain; 9 Faculty of Medical Sciences, and Health and Life, International University of Ecuador, Quito 170113, Ecuador

**Keywords:** *Mycobacterium tuberculosis*, increased sensitivity, sputum smear microscopy, TB detection, nanotechnology, infectious disease

## Abstract

A new method using a magnetic nanoparticle-based colorimetric biosensing assay (NCBA) was compared with sputum smear microscopy (SSM) for the detection of pulmonary tuberculosis (PTB) in sputum samples. Studies were made to compare the NCBA against SSM using sputum samples collected from PTB patients prior to receiving treatment. Experiments were also conducted to determine the appropriate concentration of glycan-functionalized magnetic nanoparticles (GMNP) used in the NCBA and to evaluate the optimal digestion/decontamination solution to increase the extraction, concentration and detection of acid-fast bacilli (AFB). The optimized NCBA consisted of a 1:1 mixture of 0.4% NaOH and 4% N-acetyl-L-cysteine (NALC) to homogenize the sputum sample. Additionally, 10 mg/mL of GMNP was added to isolate and concentrate the AFB. All TB positive sputum samples were identified with an increased AFB count of 47% compared to SSM, demonstrating GMNP’s ability to extract and concentrate AFB. Results showed that NCBA increased AFB count compared to SSM, improving the grade from “1+” (in SSM) to “2+”. Extending the finding to paucibacillary cases, there is the likelihood of a “scant” grade to become “1+”. The assay uses a simple magnet and only costs $0.10/test. NCBA has great potential application in TB control programs.

## 1. Introduction

One third of the world’s population carries an asymptomatic infection from *Mycobacterium tuberculosis* (*Mtb*). In 2017, 10.4 million people fell ill with tuberculosis (TB), and 1.6 million died from the disease (including 0.4 million among people with HIV) [1]. Cost, technical limitations and lack of resources make the diagnosis of TB difficult in developing countries, where the majority of cases occur.

The primary method to diagnose TB in most low and middle income countries is sputum smear microscopy (SSM), using the Ziehl-Neelsen (ZN) staining technique [2]. SSM is highly specific for *Mtb* and can identify the most infectious patients in areas where there is a high TB prevalence [2]. Although this test is easy to perform, inexpensive, provides rapid results and does not require complex laboratory equipment, it has considerable drawbacks [3]. The clinical sensitivity of this test is highly variable (20–80%) with much lower sensitivity in paucibacillary cases, such as in immunocompromised or pediatric patients where the bacterial load is usually fewer than 5,000–10,000 acid-fast bacilli (AFB) per milliliter of sputum sample [2,3,4].

The World Health Organization endorses the Xpert MTB/RIF (Cepheid, Sunnyvale, CA, USA) real-time PCR platform to diagnose TB, primarily because it can identify rifampicin resistance in a short amount of time (120 min) [5]. However, its use is limited due to the high cost (instrument, cartridges and laboratory requirements) [6]. Additionally, reports have indicated that it can provide false-positive results [7,8].

Recent advances in nanotechnology have enabled the development of new diagnostic platforms aimed at more sensitive and faster pathogen detection [9,10,11,12,13,14]. However, they are designed for operation in environments with fully supported infrastructure [10,11,12,13,14]. Simple and low-cost nanotechnology-based TB diagnostics are still needed, where there is demand for a TB diagnostic that is suitable for low-resource clinical settings and that can be easily integrated into clinical practice.

This paper reports the use of a glycan-functionalized magnetic nanoparticle-based colorimetric biosensing assay (NCBA) that can be used to capture and increase the AFB count in PTB positive sputum samples without the use of expensive and temperature-sensitive antibodies. The advantages of the assay include: (1) Room-temperature assay, (2) no need for a power supply, (3) no refrigeration, (4) affordable ($0.10/test), (5) rapid (<20 min), and (6) simple to implement. Using only a simple magnet, the glycan-functionalized magnetic nanoparticles (GMNPs) function as an extractor and concentrator of AFB without the need for an electrically powered centrifuge. No antibodies, aptamers or peptides are required. Once the GMNP-AFB complex is formed, GMNPs facilitate rapid detection due to the presence of visually observable clumped red-stained bacilli which are surrounded by brown nanoparticles. This technique could be easily integrated into conventional TB control programs in any resource-limited setting and where SSM has low sensitivity performance [15,16,17,18,19,20,21,22,23]. The assay relies on the physical (magnetic) and chemical (glycan) properties of the nanoparticles to concentrate mycobacteria cells from clinical samples. The acid-fast property of mycobacterial cells allow the color change from gray to red. Specifically, acid-fastness is a staining property shared by mycobacterial species which possess on their cell wall surface the presence of complex branched-chain hydroxy lipids termed mycolic acids, or mycolates. In the acid-fast stain, the carboxylic acid group of the mycolic acid reacts with the fuchsin dye. Subsequent application of a decolorizer will remove fuchsin from the cell walls of non-mycobacteria, but not in mycobacteria. Thus, red clumps surrounded by brown particles would indicate the presence of a mycobacterial species, hence the name “nanoparticle-based colorimetric biosensing assay” (NCBA).

## 2. Materials and Methods

### 2.1. Chemicals and Reagents

N-acetyl-L-cysteine (NALC) and sodium hydroxide (NaOH) were purchased from Sigma-Aldrich (St. Louis, MO, USA). The NaOH (0.4%) and NALC (0.025%, 1%, 2% and 4%) were prepared in distilled water. A phosphate buffer saline solution (0.01 M PBS) was prepared using standard protocols. Carbol fuchsin, 0.3%, was prepared by dissolving 50 g of phenol in 100 mL 90% ethanol and adding 3 g of basic fuchsin in the mixture which was brought to 1 L by adding distilled water. The decolorization solution was 25% sulphuric acid. The counter stain was 0.3% methylene blue. GMNPs were provided by the Alocilja Research Group from Michigan State University (USA). Briefly, GMNP (100 ± 58 nm) has a magnetite (Fe_3_O_4_) core and a glycan (chitosan) coating. Fe_3_O_4_ was synthesized using ferric chloride hexahydrate (FeCl_3_·6H_2_O) as a precursor in a mixture of ethylene glycol (as a reducing agent) and sodium acetate (as a porogen). Chitosan was polymerized to surface-modify the iron oxide nanoparticles.

### 2.2. Clinical Samples

Twenty-four left-over sputum samples collected from an equal number of patients diagnosed with PTB were used, collected from the Mycobacteriology Laboratory, TB Prevention and Control Program for the Highlands region of Chiapas, Mexico (Ministry of Health). Sputum samples (at least 1 mL) were used prior to the initiation of the anti-TB therapy. All information relating to the patients was removed and the samples were decoded prior to their handling and processing. The samples were either processed immediately on arrival at the laboratory or stored for less than 24 h at 4 °C before processing. The samples were weighed with the assumption that 1 g equals 1 mL.

### 2.3. Optimization of Digestion Reagent

Twelve of the 24 sputum samples were used to optimize digestion-decontamination. Various concentrations of NaOH and NALC were evaluated to liquefy and homogenize the samples without negatively affecting the extraction ability of GMNP. Based on previous studies in the lab (unpublished data), 0.4% NaOH was optimum and was used with different concentrations of NALC (0.025–4%) in a 1:1 ratio to liquefy and homogenize the samples. The digestion time was monitored.

### 2.4. NCBA versus SSM

**Figure 1:**
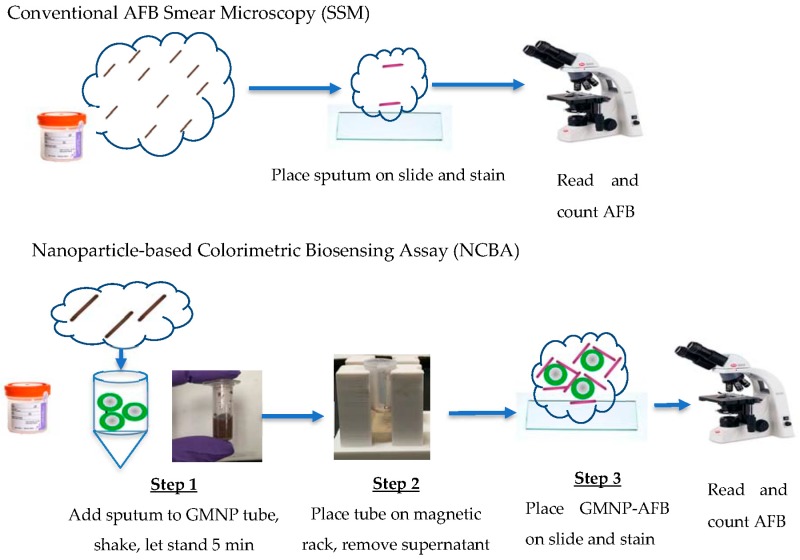
Schematic representation of sputum smear microscopy (SSM) (top) and nanoparticle-based colorimetric biosensing assay (NCBA) (bottom) approaches.

The NCBA approach was compared with the SSM method. Liquefied sputum samples were separated into two portions, one for SSM and the other for the NCBA experiments. Figure 1 shows a schematic of the SSM and NCBA approaches. The NCBA approach has two additional steps compared with SSM: The addition of GMNP to the liquefied sputum sample and magnetic separation. Unless GMNP concentration was the variable, 10 mg/mL of GMNP was used.

### 2.5. Sputum Smear Microscopy (SSM)

A smear was prepared from the first portion of the sample using the SSM method following the standard protocol [3,24]. Briefly, 20 µL of the sputum sample was placed on a microscope slide (~400 mm^2^) and heat fixed by passing flame from a Bunsen burner under the slide. The slide was then placed on a staining rack and 0.3% carbol-fuchsin was poured over the smear. The underside of the slide was gently heated by passing a flame under the rack until fumes appeared. After cooling (~2 min), the smear was rinsed with distilled water until no color appeared in the effluent, followed by washing with 25% sulphuric acid several times until the smear appeared light pink. The smear was washed with distilled water and then 0.3% methylene blue was added to cover the smear. Distilled water was used to wash off the counter stain and then the smear was air-dried. Once ready, the smear was examined under a bright field microscope (Eclipse E400, Nikon Instruments Inc., NY, USA) using a 100× oil immersion objective to observe the presence of red-colored AFB.

### 2.6. NCBA Approach

About 1 mL of the second portion of the sample was added into a 1.5 mL tube containing 0.5 mL of GMNP solution. The GMNP and sputum were mixed and allowed to incubate for 10 min at room temperature. The tube was then placed in a magnetic rack to separate the magnetic GMNP-AFB complex and the supernatant was discarded. The GMNP-AFB complexes were then washed and re-suspended in 0.5 mL of 0.01 M PBS. A smear was prepared by transferring 20 µL of the liquefied sputum sample onto a microscope slide which was processed similar to the SSM method, where it was then examined under a bright field microscope to observe the presence of any clumped red-colored AFB surrounded by brown nanoparticles.

### 2.7. Optimization of GMNP Concentration

In nine out of the 24 sputum samples, GMNP was added at three different concentrations (0.5, 10, and 20 mg/mL) to determine the best concentration for the capture, extraction, concentration and detection of AFB in the sputum samples. The subsequent procedure followed the NCBA approach which was described above. To homogenize the sputum sample, 0.4% NaOH/1% NALC was added. The time for magnetic separation was also determined.

### 2.8. Validation of the Optimized Parameters

Based on the optimization results, the remaining three of the 24 samples were used to validate 0.4% NaOH/4% NALC for digestion and 10 mg/ml GMNP for extraction.

### 2.9. Quantification of the AFB Count

The slides were mounted on a bright field optical microscope (Nikon Eclipse E400 Instruments Inc.) and examined under oil immersion (100×). One hundred microscopy fields were observed to quantify the number of AFB present in each slide, for both SSM and NCBA, and all samples per treatment of NaOH/NALC were pooled for reporting and statistical analysis.

### 2.10. Data Analysis

Statistical analysis was performed using SPSS version 21 on pooled data for each treatment, AFB counts per field and the total number of fields. Pair-wise student’s t-tests were performed to compare AFB mean counts between SSM and NCBA, in determining the digestion-homogenization treatment and the optimal GMNP concentration. Pearson’s correlation coefficient was determined to evaluate the association between the time for homogenization and magnetic separation. To measure the effect of the length of time for homogenization and magnetic separation on the number of AFB observed in NCBA, a linear regression model was used. All analyses were calculated at a 95% confidence interval (α = 0.05).

### 2.11. Capture Efficiency (CE) of GMNP for Mycobacterial Cells

To understand the dynamics of GMNP-AFB interaction, a supplemental study was conducted to determine the CE of GMNP for mycobacteria. Based on previous preliminary studies (unpublished data), 5 mg/mL of GMNP was used. Due to biosafety considerations, *Mycobacterium smegmatis* (*Msm*) was used as a surrogate for *Mtb* in the CE experiments. *Msm* shares a similar cell wall structure with *Mtb*, both are AFB species, and both can be stained using the ZN technique [25,26]. Artificial sputum was prepared following the polyacrylamide-based artificial sputum method [27]. A *Msm* culture was grown in a MiddleBrook 7H9-ADC broth and incubated until the optical density reached 0.6 at 600 nm (about log phase). Serial dilutions of the *Msm* bacteria were prepared and spiked into the artificial sputum, followed by incubation for 10 min at room temperature along with manual shaking. The GMNP-*Msm* complexes were magnetically separated using a simple magnetic rack and the supernatant was removed. The complexes were washed twice and re-suspended in 0.5 mL of 0.01 M PBS. The separated complexes along with pure bacterial dilutions were plated on a MiddleBrook 7H10-ADC agar and incubated for seven days to allow the growth of *Msm* cells. The CE was determined as the logarithm of cells captured divided by the logarithm of cells in the original dilution, as in Equation (1):

(1) $$CE=\frac{\log\left(cell\;count\;of\;cells\;captured\right)}{\log\left(cell\;\mathrm{count}\;\mathrm{of}\;\mathrm{cells}\;\mathrm{in}\;\mathrm{original}\;\mathrm{dilution}\right)}$$

### 2.12. Transmission Electron Microscope (TEM) Imaging

GMNP-*Msm* complexes captured according to the previous section were visualized using a JEOL 100 CX transmission electron microscopy (TEM) at the MSU Center for Advanced Microscopy with a magnification range of 5000× to 80,000×.

### 2.13. Ethics

Human subjects: Informed consent for participation was obtained from the patients and ethical approval was obtained from the Research Committee at ECOSUR (CEI-O-47/14) as well as from the Ministry of Health for Chiapas (5003/5342), under the project “Testing and evaluating a low-cost, field-operable biosensor for rapid detection of pulmonary tuberculosis”. Sputum samples were used only for the purpose of this study and the remaining samples were disposed according to biosafety guidelines.

## 3. Results

### 3.1. Effect of NaOH-NALC Treatments on AFB Count

The role of NaOH is to eliminate the normal upper respiratory tract flora and the role of NALC is to break down the disulfide bonds in mucin so that the sputum is liquefied and the AFB are released during the assay. The AFB count using NCBA increased to 35% with 1% NALC, followed by 22% with 2% NALC, and finally by 143% with 4% NALC (1216, 9353, and 7682, respectively, Table 1) for the same number of fields counted compared to SSM (903, 7677, and 3160, respectively) for pooled data of 300 fields for each treatment (three samples per treatment, total of 12 samples). NALC at 4% produced the highest AFB count.

### 3.2. Effect of GMNP Concentration on AFB Count

For the samples tested, GMNP at 10 mg/mL was the best concentration, with an AFB count of 2575 compared to the SSM with 1958 (an increase of 32% using NCBA). For 20 mg/mL GMNP, significant agglomerations were observed on the smear slides that affected the ability to clearly count the cells. GMNP at 0.5 mg/mL had a negative effect on the AFB count for NCBA, potentially due to reduced magnetic extraction effect while increasing the drag factor (Table 2).

### 3.3. Validation of the Optimized Parameters

**Figure 2:**
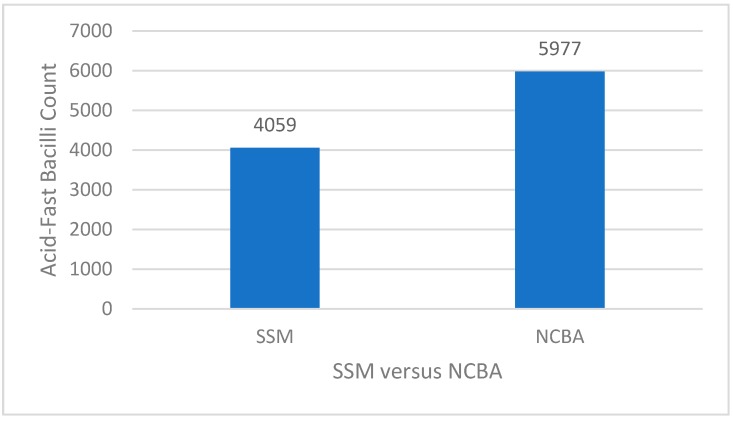
Results of the optimized parameters using 0.4% NaOH/4% NALC and 10 mg/mL glycan-functionalized magnetic nanoparticles (GMNP), with NCBA increasing AFB count by 47% compared to SSM.

Based on the optimization results (Table 1 and Table 2), the optimized treatment was 0.4% NaOH/4% NALC in a 1:1 ratio and 10 mg/mL of GMNP. Figure 2 shows that by using the optimized parameters, the AFB count for SSM was 4059 and NCBA was 5977, increasing NCBA by 47%, showing the extraction and concentration effect. The clinical relevance of this result is that an AFB count below 5000 in SSM would be considered a paucibacillary case and would likely lead to a false negative result in conventional settings, increasing the risk of TB transmission. With NCBA, the AFB count is close to 6000, increasing the chance of a positive result and getting treatment, thus minimizing transmission while increasing TB control.

### 3.4. Effect of NCBA in Grading Smears

Each of the 24 samples was graded according to standard guidelines by a trained microscopist: Seven were graded as 1+ and 17 were graded as 3+ (Table 3). The pooled AFB count for 1+ was 685 and 977 for SSM and NCBA, respectively, showing a gain of 43% by NCBA. The pooled AFB count for 3+ was 28,504 and 35,344 for SSM and NCBA, respectively, showing a gain of 24% by NCBA. Table 3 shows that 1+ would upgrade to 2+ using the NCBA. The equivalent AFB count per mL (20 µL of sample on 100 fields) was calculated and is also presented in Table 3. This result is impactful as there are many clinical cases of low bacillary counts (paucibacillary).

**Figure 3:**
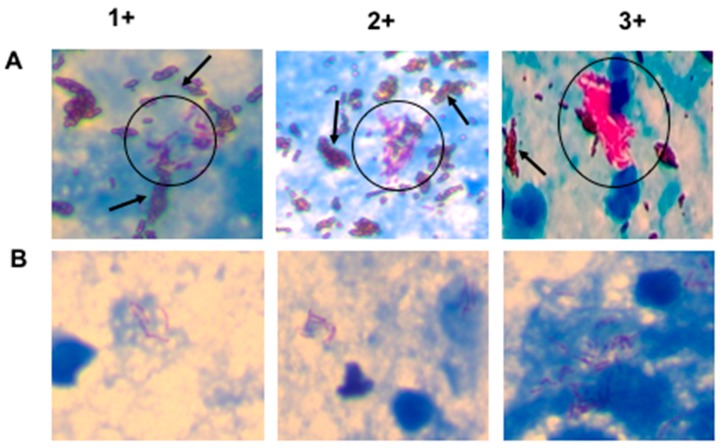
Images of AFB cells on smear slides for NCBA (**A**) and SSM (**B**). AFB appear dark red, surrounded by brown nanoparticles in NCBA. Representative images of sputum samples graded as 1+, 2+, and 3+ are shown. There is an obvious agglomeration of GMNP-AFB in the NCBA. The blue background comes from the methylene blue that is added during the assay to decolorize non-mycobacterial cells.

Figure 3 shows images of stained AFB slides for SSM and NCBA on sputum samples graded as 1+, 2+, and 3+, according to the guidelines by the WHO and IUATLD [24]. There is an obvious concentration effect using NCBA (Figure 3A) compared with SSM (Figure 3B) and also a higher AFB count in NCBA, compared to SSM. In some fields, the number of AFB observed in NCBA were highly clustered.

SSM positivity and grade indicate relative bacterial load, associated disease presentation, and infectivity for patients with TB according to the guidelines by the International Union Against Tuberculosis and Lung Disease (IUATLD) and the World Health Organization (WHO). The grade is either 3+, 2+, 1+, scanty or negative, according to the number of AFB present in one hundred microscopic fields. A single sputum sample must have between 5000–10,000 cells per mL for reliable TB detection; below this concentration, SSM might give either negative or uncertain result.

### 3.5. Effect of Homogenization and Magnetic Separation on AFB Count

Statistical analyses were conducted to assess whether the sample volume, time for homogenization and time for magnetic separation influenced AFB count in the NCBA. The results showed that there was no association between the sample volume and the mean time of homogenization (Pearson’s correlation, −0.016; *p* = 0.42), implying that sample volume did not affect homogenization time. However, the mean time for homogenization and magnetic separation were correlated (Pearson’s correlation, 0.643; *p* < 0.001). The longer it took to homogenize the sample, the more time was needed to magnetically separate the GMNP-AFB complex from the rest of the sputum matrix. It was also found that a longer period of homogenization resulted in higher AFB counts, while longer magnetic separation time decreased the number of AFB observed per field in NCBA (Global correlation coefficient, 0.187, *p* < 0.001). The long homogenization times are likely due to the high mucopurulence of the sputum samples, which is related to high viscosity and high cellular load. Highly viscous samples reduce the mobility of GMNPs in the matrix and the formation of GMNP-AFB complexes, which can have an influence on their magnetic separation.

### 3.6. Supplementary Study Using Msm

**Figure 4:**
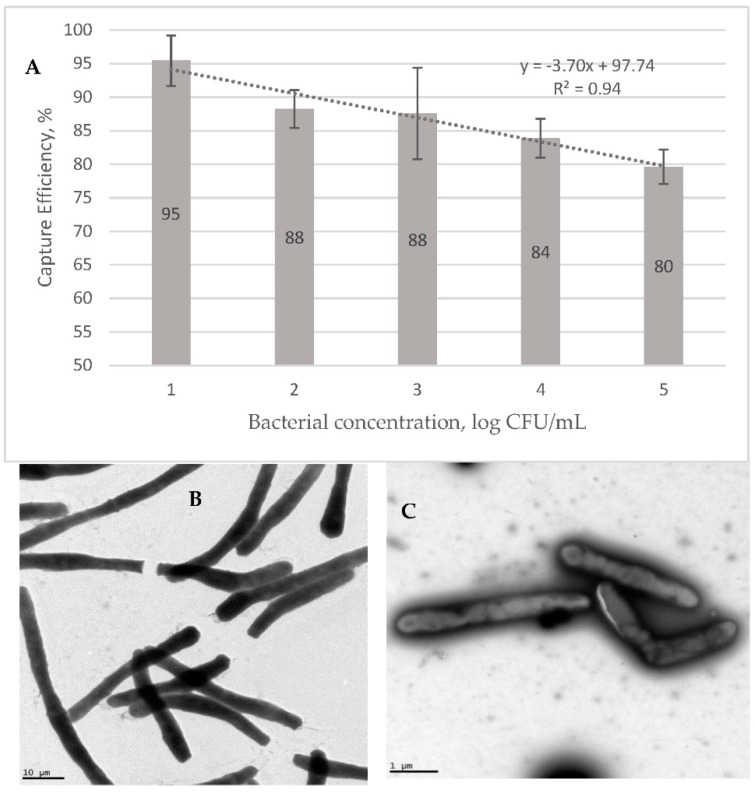
(**A**) Capture efficiency of GMNP for extracting *Msm* from artificial sputum. TEM images of (**B**) corded *Msm* without GMNP and (**C**) corded *Msm* with GMNP attached to specific site on the cell wall.

Figure 4A shows the capture efficiency (CE) of the GMNP in different concentrations of *Msm*. These were prepared in artificial sputum, magnetically extracted and plated on a MiddleBrook 7H10-ADC agar. The data shows that the CE decreases linearly with increasing bacterial concentration, from 95% at 10^1^ CFU/mL to 80% at 10^5^ CFU/mL. This result supports the sputum data in Table 3, where 1+ samples transform to 2+ using NCBA, since a lower bacterial concentration would result in a higher CE. Figure 4 also shows TEM images of corded *Msm* without GMNP (B) and with GMNP (C).

## 4. Discussion

SSM continues to be the main TB diagnostic method in medium and low income countries due to its affordability, simplicity in process and rapid results. However, its sensitivity ranges from 20–80%, mainly due to the high AFB cell concentration that is required (5000–10,000 cells/mL). Different chemical and physical sputum processing methods have been studied to improve its sensitivity, but standardization of the treated samples is among major drawbacks [28]. The use of antibodies and peptides for bacterial extraction in a matrix is a common practice. In a study by Foddai et al. [29], magnetic beads coated with specific peptides yielded an 85–100% CE when used to separate *Mycobacterium avium* subsp. *paratuberculosis* from broth samples containing 10^3^–10^4^ cfu/mL of the bacteria. Roberts and Hirst [30] used antibodies against *Mycobacterium ulcerans* to isolate the bacteria and yielded a 70–85% CE.

Studies evaluating nanoparticles synthesized with diverse materials have used spiked sputum samples from healthy donors and sophisticated equipment to detect whole mycobacterial cells [12,31,32,33]. Those studies have reported good detection of mycobacteria, but the assays are complex and expensive.

Sputum is a complex specimen consisting of linked mucin molecules, filamentous actin, cell debris, DNA, leukocytes, proteoglycans, inflammatory mediators and elastin fibers [34]. Embedded within this matrix are bacteria including *Mtb* in TB+ sputum. *Mtb* cells clump due to cording within the specimen, resulting in an unequal distribution. The efficient release of bacteria from sputum specimens requires a chemical and/or mechanical breakdown of the linked mucin and actin molecules [30]. For this study, a combination of NaOH (0.4%) and NALC (0.025–4%) in a 1:1 ratio was explored for use in sputum digestion. The rationale for testing low and high concentrations of NALC was to determine if the solution would affect the overall performance of NCBA. Higher NALC concentration results in better liquefaction of the sputum and subsequently better extraction of AFB, however, that would also mean a higher cost since NALC is expensive. The assay is designed as a low-cost system for application in resource-limited environments. The results show that 0.4% NaOH/4% NALC yielded the best performance, as indicated by the higher AFB count in NCBA, with an overall 143% increase over SSM (Table 1). At a lower concentration of NALC (0.025%), SSM resulted in higher AFB counts compared to NCBA, potentially due to the sputum not being fully liquefied and the GMNP-AFB complexes not being able to be released from the clumped matrix.

The use of a glycan bioreceptor on GMNP allows for simple and inexpensive capture of Mtb cells in the mucoid sputum samples without the use of expensive antibodies or aptamers. The Mtb cell envelope is particularly rich in complex carbohydrate-containing molecules, such as glycolipids (e.g., phosphatidyl-myo-inositol mannosides), lipoglycans (e.g., lipomannan and lipoarabinomannan), polysaccharides (e.g., α-glucan) and glycoproteins. These mycobacterial carbohydrate molecules can bind with the glycan functionalized nanoparticles. Furthermore, cold storage is not required, allowing its application in conditions without refrigeration and electricity.

GMNP at 10 mg/mL was found to be the optimal amount to capture and concentrate AFB which resulted in the highest AFB count per field (Table 2, Figure 3). At a higher GMNP concentration (20 mg/mL), smears were difficult to observe due to high aggregation. 

NALC at 1% and 2% resulted in 35% and 22% increases in AFB count, respectively. NALC at 4% resulted in a 143% increase in AFB count. If an input/output ratio is calculated, that is, %NALC/%AFB increase, 1/35 and 4/143 each equals to 0.028 while 2/22 equals to 0.09. This shows that increasing NALC concentration to homogenize the sputum sample correspondingly increases the AFB count, except for the 2% NALC that is more of an outlier. The experiments used 4% NALC as the upper value to minimize cost. It would be interesting to determine in future experiments the upper limit of NALC.

Also, longer homogenization time was correlated with longer magnetic separation. This could be due to the viscosity of the sputum which would then slow down the magnetic separation. Long magnetic separation had a negative effect on the AFB count. The longer the magnetic separation, the longer the exposure of AFB to the NaOH/NALC homogenization solution, which would then increase the exposure of the sample to the decontamination effect of NaOH, subsequently reducing the viability of the AFB.

### Determining GMNP’s Capture Efficiency for Mycobacterial Cells

Results from the GMNP extraction experiments using *Msm* showed that the CE ranged from an average of 80% to 95% for samples with AFB concentrations ranging from 10^1^ cfu/mL (n = 3) to 10^3^ cfu/mL and 10^4^ cfu/mL (n = 10 each) (Figure 4A). The negative linear relationship between bacterial cell concentration and the CE indicates that NCBA would be highly useful in cases of low bacterial load, as in paucibacillary cases. These data show that as cells increase, CE decreases, potentially due to epitope competition. The lower the number of bacteria in the solution, the higher the ratio of nanoparticles is to bacterial epitopes, that is, more nanoparticles are available to bind and self-assemble on the surface of a bacterium. As the number of bacteria in the solution increases, the resulting higher number of bacterial epitopes compete with the available number of nanoparticles in the solution. Such a nanoparticle-based bacterial detection system would be highly sensitive at lower cell concentrations.

The physical interaction between GMNP and the *Msm* cell wall was also confirmed using TEM (Figure 4B,C). GMNPs attach to specific sites on the cellular surface through the carbohydrate-binding lectins on the bacterial surface. These data show that the GMNP interacts with the mycobacterial cell wall, forming a complex.

## 5. Conclusions

In summary, these results show that the NCBA technique has significant potential application in improving the diagnosis of paucibacillary TB cases (e.g., in immunocompromised individuals, children, drug-resistant TB, among others). Due to its ability to concentrate AFB, NCBA could improve one grade higher, making an initial case at 1+ to become 2+ with the assay, or go from “scant” to become 1+ (Table 3). The assay uses only a simple magnet and does not require any major equipment, electricity or refrigeration, can be completed in 10–20 min and only costs $0.10 per test. The glycan-based magnetic isolation method is novel and has advantages due to its affordability, long shelf-life, room-temperature storage and easy preparation. Thus, this system can be easily incorporated in clinical practice to enhance TB diagnosis, especially in low resource environments. It has been hypothesized that a rapid and universally accessible TB test with a sensitivity of 85% and specificity of 97% has the potential to save close to 400,000 adjusted lives annually, or 27% of the global TB deaths [35]. This NCBA technology satisfies the criteria well for rapid, affordable and universal access.

Future work will include determining the limit of detection (LOD) and comparing NCBA with cultures on solid media (7H11 Middlebrook), leading to sensitivity evaluation in paucibacillary cases. The assay will also use blinded samples in comparison with SSM, not only in sputum samples, but also in gastric samples from pediatric patients.

## Figures and Tables

**Table 1:** Optimization of N-acetyl-l-cysteine (NALC) treatment.

Concentration of NALC Solution	Number of AFB by SSM	Number of AFB by NCBA	No. Fields Observed	% Increase (NCBA-SSM)
0.025%	6525	3958	300	−39%
1%	903	1216	300	35%
2%	7677	9353	300	22%
4%	3160	7682	300	143%

Abbreviations: AFB, acid-fast bacilli; SSM, sputum smear microscopy; NCBA, nanoparticle-based colorimetric biosensing assay.

**Table 2:** Optimization of the glycan-functionalized magnetic nanoparticles (GMNP) concentration.

Concentration of GMNP	Number of AFB by SSM	Number of AFB by NCBA	No. Fields Observed	% Increase (NCBA-SSM)
0.5 mg/mL MNP	876	391	300	−55%
10 mg/mL MNP	1958	2575	300	32%
20 mg/mL MNP	3979	5169	300	30%

Abbreviations: AFB, acid-fast bacilli; SSM, sputum smear microscopy; NCBA, nanoparticle-based colorimetric biosensing assay.

**Table 3:** Acid-fast bacilli (AFB) count analysis of the graded samples.

	1+ Grade ^a^	2+ Grade ^a^	3+ Grade ^a^
**SSM, Total AFB counts**	685		28,504
**NCBA, Total AFB counts**	977		35,344
**No. Fields**	700		1700
**Percent increase by NCBA**	43%		24%
**SSM, AFB per 100 HPF**	98		1677
**NCBA, AFB per 100 HPF**	140	140	2079
**SSM, AFB/mL**	4.9 × 10^3^		8.4 × 10^4^
**NCBA, AFB/mL**	7.0 × 10^3^		1.0 × 10^5^

Abbreviations: SSM, sputum smear microscopy; NCBA, nanoparticle-based colorimetric biosensing assay; HPF, high power fields. ^a^ Based on WHO and IUATLD grading scale: Negative (0 AFB/100 HPF); Scanty (1–9 AFB/100 HPF); 1+ (10–99 AFB/100 HPF); 2+ (100–9900 AFB/100 HPF on average); 3+ (>10,000 AFB/100 HPF on average).
